# Bacterial Therapy of Cancer: A Way to the Dustbin of History or to the Medicine of the Future?

**DOI:** 10.3390/ijms24119726

**Published:** 2023-06-03

**Authors:** Larisa N. Ikryannikova, Neonila V. Gorokhovets, Darya A. Belykh, Leonid K. Kurbatov, Andrey A. Zamyatnin

**Affiliations:** 1 Institute of Molecular Medicine, Sechenov First Moscow State Medical University, Trubetskaya 8/2, 119991 Moscow, Russia; larisa.ikryannikova@gmail.com (L.N.I.); gorokhovets@gmail.com (N.V.G.); darya-belyx@mail.ru (D.A.B.); 2 Orekhovich Research Institute of Biomedical Chemistry, Pogodinskaya 10/8, 119991 Moscow, Russia; kurbatovl@mail.ru; 3 Faculty of Bioengineering and Bioinformatics, Lomonosov Moscow State University, Leninskie Gory 1/73, 119234 Moscow, Russia; 4 Belozersky Research Institute of Physico-Chemical Biology, Lomonosov Moscow State University, Leninskie Gory 1/40, 119992 Moscow, Russia; 5 Scientific Center for Translation Medicine, Sirius University of Science and Technology, 1 Olympic Ave, 354340 Sochi, Russia

**Keywords:** bacteria-mediated tumor therapy, tumor-targeting bacteria, tumorigenic effect of bacteria, tumor microbiome

## Abstract

Bacteria are the constant companions of the human body throughout its life and even after its death. The history of a human disease such as cancer and the history of microorganisms, particularly bacteria, are believed to closely intertwined. This review was conceived to highlight the attempts of scientists from ancient times to the present day to discover the relationship between bacteria and the emergence or development of tumors in the human body. Challenges and achievements of 21st century science in forcing bacteria to serve for cancer treatment are considered. The future possibilities of bacterial cancer therapy, including the creation of bacterial microrobots, or “bacteriobots”, are also discussed.

## 1. Introduction

The history of a human disease such as cancer and the history of microorganisms, particularly bacteria, are closely intertwined. The relationship between bacterial infections and the treatment of “swellings”, which were quite probably the cancerous tissue, have been documented by the Egyptian physician Imhotep more than four thousand years ago [1,2]. Some observations describing the facts of regression of a malignant tumor in people exposed to bacterial infection were mentioned in medical documents of the early 19th century [3]. On the other hand, the infectious theory of carcinogenesis, which linked the occurrence and spread of tumors to bacterial or parasitic infection, was popular throughout the late 19th century and the first third of the 20th century. Many microbiologists have tried to find a microbial agent that causes cancer and prove its role in oncogenesis. Near the same time, the first anticancer “vaccines” were developed, based on inactivated or weakened bacteria. However, the results were poorly reproducible, while the vaccines were not universal (one “vaccine” could help against a certain type of cancer but was useless against another). As a result, the theory of bacterial (parasitic) oncogenesis was generally rejected by the scientific and medical communities to the middle of the 20th century. Despite this, publications have continued to occur only sporadically until recently, when interest in this topic appeared again due to the numerous reports establishing the connection of some types of cancer with bacterial agents [4,5,6,7,8,9,10,11,12]. *Helicobacter pylori* was the first bacterium whose etiological role in gastric cancer was proven.

Today, the burden of infections that are associated to human cancer is steadily increasing. The International Agency for Research on Cancer (IARC) estimated that one-in-five cancer cases worldwide are caused by infection [13].

Having become interested in this topic, the authors initially planned to write a thorough retrospective review including the little-known investigations of scientists from Russia (namely A.S. Troitskaya and V. A. Krestovnikova, see below). However, as the review was being written, it became clear that the future of bacterial cancer therapy is much more interesting than its past. Therefore, the main purpose of this review became to create for the readers a bridge from the first doctors and scientists searching for a “cancer microbe” to researchers of the 21st century designing a bacteriobot. Unfortunately, since the volume of the article is not unlimited, we can only provide a quick look at current trends and achievements in this field of science without claiming to be exhaustive.

## 2. Bacterial Dualism: What Can Kill Can Also Heal

### 2.1. In Pursuit of the “Cancer Germ”

Numerous data indicating a certain role of bacteria in the origin and pathogenesis of malignant neoplasms have been repeatedly obtained. Beginning in the mid-19th century, microbiologists examining tumor tissues microscopically discovered in them a variety of microorganisms [14]. Additionally, although the 150-year-old research can be viewed with some skepticism due to the lack of sterility at that time, more recent works probably deserve more attention. By the beginning of the 20th century, the principles of asepsis were generally defined and increasingly introduced into medical and surgery practice—this reduced the chance that the microorganisms observed by researchers in surgery tumors were introduced from the outside. Thus, in the works of Sanfelice (1895), Plimmer (1899), Robertson (1921), and Rappin (1934), some microorganisms observed in tumors of various etiologies were described. In some cases, these microorganisms were successfully cultured on special media, and when trying to introduce a culture of these microorganisms to animals, tumors occasionally developed [14,15,16]. In 1885, a French scientist, T. Doyen, isolated from the different tumors a bacterium that he named *Micrococcus neoformans*. This microorganism had an extremely small size (about 0.13 microns) and was located in the tumor tissues both intracellularly and in the intercellular space. When inoculated into animals, it “gave rise to neoplastic formations” [14,15,17]. However, subsequent studies have not been able to confirm the etiological significance of “*Micrococcus neoformans*” in the pathogenesis of malignant tumors [17]. Dudgeon and Dunkley noted that this microorganism was similar and may be related to the staphylococcal species [17]. Many researchers have noted the pleomorphism of microorganisms isolated from tumors. Thus, in 1930, T.J. Glover obtained from an adenocarcinoma of the human breast an intracellular organism that was shown in subculture to be highly pleomorphic: its lifecycle included coccoids, rods, mycelial stages, and filter-passing forms. The introduction of a culture of this microorganism to dogs, guinea pigs, rats and mice, as well as monkeys, in some cases led to the appearance of tumors in them [15]. Three-quarters of a century later, a work was published suggesting that “Glover’s organism” might be the highly pleomorphic bacterium *Bacillus licheniformis*; however, this idea was not evolved [18]. Similar observations of pleomorphic microorganisms can be found in the works of J. Nuzum (1925) [19], F. Blumenthal (1927) [20], and V. Livingston-Wheeler (1947) [14,15]. Thus, J. Nuzum cultured Gram-positive micrococci from breast tumors. These cocci were facultative anaerobes; they did not grow on solid media, but could be successfully cultured on ascitic fluid. Their size varied from 0.1 to 0.3 µm depending on the growth stage, and larger forms similar to staphylococci appeared in older cultures. Microscopic studies have shown that these microorganisms can be discovered both in the intercellular fluid and in the cytoplasm of tumor cells. Inoculation with this bacterium into mice and dogs caused the development of some precancerous lesions and, in some cases, mammary carcinomas [14,19]. V. Livingston-Wheeler, together with R. Allen, E. Alexander-Jackson, and other scientists, found that there were microorganisms in malignant tumors of different histogenesis that could be isolated and cultured. Histological and microscopic studies have shown that this “mycobacterium like” specific microorganism (called “*Mycobacterium tumefacience*”) is extremely polymorphic and can represent mobile rods, cocci, or the smallest filterable forms [21]. Mobile rods were easily cultured on conventional media, while the growth of small filterable forms was achieved only on special media. Different forms of the microorganism were differently Gram-stained [14]. Later, taxonomic studies did not confirm that this bacterium belonged to the mycobacteria group, so it was renamed “*Progenitor cryptocides*” [21,22]. However, it was not possible to convincingly demonstrate experimentally the etiological role of this microorganism in the occurrence of malignant tumors—granulomas appeared in infected animals, but neoplastic transformation did not occur.

### 2.2. Scientists Make Bacterial “Anti-Cancer Vaccines” Almost Blindly, and It Sometimes Works!

Simultaneously with the search for a “cancer germ”, the attempts began using microorganisms to treat oncopatients. One of the probably first cases was described in the 1860s by German physician W. Busch, when a patient with a cancer of unknown etiology was intentionally infected by transferring her into a bed in which another patient had died from erysipelas, an infection caused by *Streptococcus pyogenes*. As expected, the patient became infected, and the tumor started to regress; however, the patient soon also died of infection [1,23,24]. Other of the most famous in this area were Coley’s experiments of the early 20th century, who observed that, when patients with sarcomas had acute streptococcal infections, their tumors regressed. He developed a “vaccine” (“Coley’s toxins”) composed of two killed bacterial species, *Streptococcus pyogenes* and *Serratia marcescens*, to simulate an infection with the accompanying fever without the risk of an actual infection [25,26,27]. As a result, prolonged or even complete regression of advanced malignancy was documented in some cases [1,23,28]. Moreover, mentioned above, T. Doyen tried to produce an anticancer “vaccine” from the “*Micrococcus neoformans*” he discovered [17]. Livingston-Wheeler also produced an autologous vaccine utilizing her “*Progenitor cryptocides*” organism for the treatment of cancer patients. It was even reported that her husband, who developed a malignant lymphoma of the neck, was healed with the help of this vaccine in six months [15]. Among others, the work of A.S. Troitskaya deserves to be mentioned, who microscopically examined the blood of patients with various types of malignant tumors in the 1960s of the 20th century, and observed some spherical formations (she called them “globoid bodies”) which were not found in the blood of healthy people. These bodies were located both inside and outside the white blood cells; moreover, their number correlated with the severity of the cancer process. Further experiments with “globoid bodies” allowed her to grow on special media some polymorphic microorganism whose etiological linkage with malignant neoplasms was suggested [29,30]. Similar observations were made around the same time by V. A. Krestovnikova [31]. Later, this microorganism was identified as a particular species of corinemorphic bacterium and was named Corinebacterium Krestovnikova–Troitskaya. Based on isolated hemocultures, Troitskaya began the production of anticancer “autovaccines”, the application of which provided good results in some cases [14]. In 1995, an immunomodulatory pharmaceutical drug on the basis of the Corinebacterium Krestovnikova–Troitskaya strain, which demonstrated a good activity against various types of neoplasms, was patented [32,33].

The studies mentioned above are just a few of a quite large number of works devoted to the microbiological study of malignant tumors. Unfortunately, neither all nor even most of these works can be covered even briefly in this short retrospective section. In general, the results of these works can be summarized as follows: (1). First, it is a fact that many researchers observed some microbes in human or animal tumors or the blood of cancer patients. These bacteria isolated from tumor tissues were often highly pleomorphic, i.e., they showed a marked degree of morphological variation, one phase of which was often filterable (i.e., able to pass through a submicron membrane filter). (2). The taxonomic affiliation of these microorganisms has often not been established correctly, so now it is difficult to understand what species it belonged to from the point of view of current modern classification. (3). Being injected into animals, these microbes in some cases could cause the development of malignant neoplasms, while in others they did not. (4). There have been attempts to create “anti-cancer vaccines” using these microbes, and there are confirmed cases where it helped to fight the disease or improve the patient’s quality of life. (5). Unfortunately, it is always questionable: did the microbes observed by the researchers actually exist inside the tissue, or was their presence the result of insufficiently sterile manipulation with tissues?

The information accumulated for more than a hundred years had remained almost unclaimed when two events occurred at the end of the 20th century. First, the etiological role of *Helicobacter pylori* was established—or, rather, officially recognized—in gastric cancer development. Secondly, researchers developed an incredible molecular biological toolkit, including the possibility of studying the taxonomic composition of microbial communities even without bacteria culturing. The latter provided a chance to draw closer to understanding the microbiome of malignant tumors.

## 3. Tumor Microbiome: Peaceful Inhabitants or Malicious Instigators?

Technological advances in DNA sequencing have provided unprecedented insights into the composition of the microbiomes, in particular of different parts of the human body, including malignant neoplasms [34]. Here, we should note that the study of the tumor microbiome is a quite difficult task. First of all, it should distinguish the tumors originating on mucosal surfaces that either harbor a diverse microbial community (microbiota) or are routinely exposed to microbes from the environment (i.e., naso- and oropharynx, esophagus, gastrointestinal tract, or skin) from tumors in body sites that are typically considered sterile (the lung, liver, bladder, bone, blood, etc.). In the first case, it is necessary to detect a probably tiny difference in the composition of the tumor microbiome from that of the surrounding tissues, while in the second case, to be sure that there is no possible outside contamination introduced at the time of extraction or while manipulating a tumor tissue. This is of particular importance because the tumor microbiome was established to have a relatively low biomass. In the very recent and excellent work of D. Nejman et al. [35,36], which is the most rigorous and comprehensive survey of bacteria in human tumor samples to date [37,38], the microbiome of 1526 samples from seven human tumor types (breast, lung, melanoma, pancreas, ovary, bone, and glioblastoma multiforme) was characterized. Authors used hundreds of negative controls and created a series of computational filters to remove the traces of any bacteria that could have come from outside the tumor samples. It was discovered that every single cancer type harbored metabolically active (live) bacteria, and that different cancer types harbor different bacteria species. The highest number and diversity of bacteria was revealed in breast cancers. Moreover, many more bacteria can be found in breast tumors compared to the normal breast tissue surrounding these tumors. Electron microscopy visualization of these bacteria demonstrated that they prefer to locate in specific places inside the cancer cells—close to the cell nucleus. Possibly, the bacteria may live off certain metabolites that are overproduced by or stored within the specific tumor types.

Thus, the observations of many scientists who had been reporting the presence of certain microbes inside the tumor and tumor cells for more than a hundred years can be considered convincingly confirmed. However, where do microorganisms come from inside a “sterile” tumor? It can be assumed that, as tumors develop, their disorganized, leaky vasculature may allow circulating bacteria to enter, and the immunosuppressed environment as well as the presence of nutrients within the hypoxic region may provide a safe haven for them. Intratumor bacteria may also arise from the normal adjacent tissues if the latter are typically a place of their colonization.

However, even if bacteria do live inside tumors and tumor cells, the main and most interesting question—whether these bacteria are directly involved in the process of oncogenesis—still remains unanswered. The authors emphasize that there is no evidence that the microbiome or any specific taxa *cause* cancer, at least outside of other factors of carcinogenesis [35]. Therefore, in the next section, we will consider cases where the link between bacterial infection and tumor development are believed to be established.

## 4. Tumorigenic Effect of Bacteria: Microbes, Which Are Typically Linked to Cancer, and Probable Mechanisms of Carcinogenic Action

Before discussing the intriguing question of the role of bacteria in carcinogenesis, it is worth clarifying what it means to participate in carcinogenesis. The classification of carcinogenic agents was developed by the International Agency for Research on Cancer based on a set of precise and strict criteria, including the genotoxicity of the potential carcinogenic agent, its electrophilicity and immunosuppressivity, the ability to alter DNA repair or cause genomic instability, induce epigenetic alterations, oxidative stress, or chronic inflammation, to modulate receptor-mediated effects, cause immortalization, and alter cell proliferation, cell death, or the nutrient supply [39,40]. According to these criteria, only one bacterial pathogen is defined to date by the IARC as a human carcinogenic agent—*Helicobacter pylori*, which is considered as the strongest known risk factor for gastric cancer [41,42,43,44]. The researchers who showed its role in inducing stomach ulcers were awarded the Nobel prize in 2005. According to estimations, more than half the world’s population is infected with *H. pylori*; however, to be fair, only a small minority (1–2%) of those infected develop gastric cancer or precancerous gastric lesions.

By colonizing the stomach, *H. pylori* can cause chronic inflammation and induce high levels of expression of proinflammatory cytokines, which cause oxidative stress and oxidative DNA damage in the infected mucosa. On the other hand, increased production of free radicals—reactive oxygen/nitrogen species (ROS/RNS)—near *H. pylori* takes place, leading to an increase in the mutation rate of host cells. Altogether, this leads to the genomic instability and activates oncogenic pathways. The more or less virulent strains of *H. pylori* were described: those strains that produce high levels of proteins such as vacuolating cytotoxin A (VacA), cytotoxin-associated gene A protein (CagA), and neutrophil-activating protein A (NapA) appear to cause more tissue damage than those that produce lower levels or are completely devoid of these proteins. Another effect of the CagA protein is suggested to prevent the human immune system from recognizing and killing bacterial cells, which is a critical factor in the long-term survival of this bacterium inside the stomach. *H. pylori* also causes a variety of epigenetic changes which can be associated with the development of cancer. These epigenetic changes occur due to the *H. pylori*-induced methylation of CpG sites in gene promoters and the altered expression of multiple microRNAs. Epigenetic changes lead in particular to a decrease in the effectiveness of the DNA repair mechanism, contributing to the accumulation of mutations and genomic instability. The development of gastric carcinoma is a step-by-step process, and is typically preceded by the development of chronic gastritis, intestinal metaplasia, and dysplasia [45]

**Figure 1:**
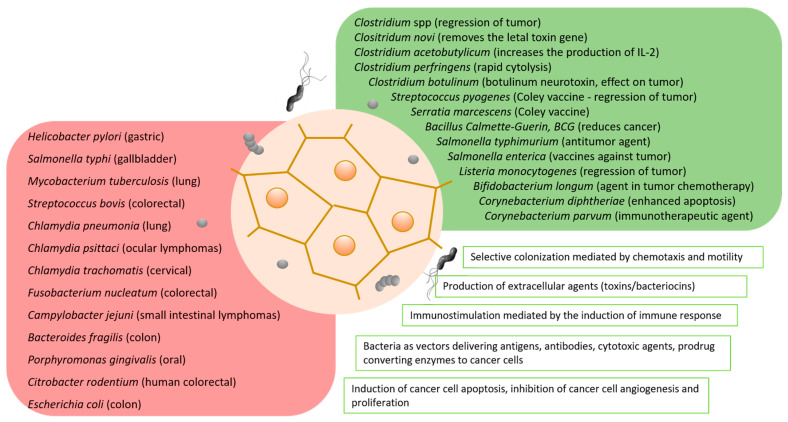
Bacterial species that are commonly discussed as associated or even being causative agents of cancer, and bacteria that were attempted to be used to produce drugs for the treatment of cancer patients, or at least to improve the well-being of patients.

**Figure 2:**
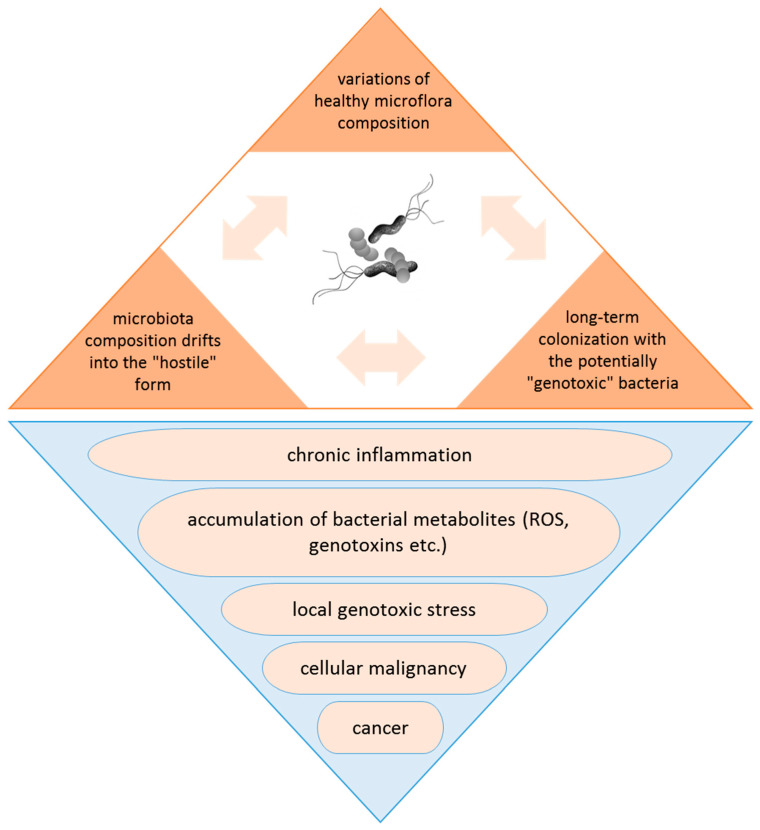
Changes in the taxonomic composition of the bacterial microbiota leading to the development of the disease. However, perhaps conversely, the disease causes a transformation of the microbiota composition, shifting it to the “unfriendly”, hostile form.

In addition to *H. pylori*, there are several other bacterial species that are commonly discussed as associated or even being causative agents (triggering factors?) of cancer (Table 1). Among them are *Salmonella typhi*, which is considered as linked to hepatobiliary carcinoma and gallbladder cancer, *Campylobacter jejuni* (small intestinal lymphomas), *Chlamydia psittaci* (ocular lymphomas), *Mycobacterium tuberculosis* and *Chlamydia pneumonia* (lung cancer), *Fusobacterium nucleatum*, *Bacteroides fragilis*, *Streptococcus bovis* and *Citrobacter rodentium* (human colorectal cancer), *Chlamydia trachomatis* (cervical cancer), *Porphyromonas gingivalis* (pancreatic and oral cancer), and some others (Figure 1). These bacteria are usually found in the altered microbiota of the damaged tissues of cancer patients. There are many investigations demonstrating that these bacteria secrete virulence factors resulting in oxidative stress, chronic inflammation, and host DNA damage that can promote carcinogenesis. It is still unclear whether quantitative or qualitative changes in the taxonomic composition of the bacterial microbiota caused by the harmful effects of the environment lead to the development of the disease, or, conversely, the disease causes a transformation of the microbiota composition, shifting it to the “unfriendly”, hostile form. However, the long-term colonization of the tissue by the bacterial species that are able to cause local genotoxic (DNA-damaging) stress can lead to cellular transformation and ultimately to cancer development (Figure 2) [7,45,46,47].

## 5. Bacteria in Cancer Therapy

Referring to the previous chapter, it is all the more surprising that bacteria can also be considered as therapeutic tools against cancer [46,64,65,66]. Even when the role of bacteria in oncological processes was not very clear, an attempt was made to use them to treat or at least relieve the sufferings of oncologic patients—and sometimes it worked, as mentioned in Section 2. Today, much more is known about the possible ways in which bacteria can affect the development of tumors [67,68,69]. Currently, one of the main and generally accepted versions of the beneficial effect of bacterial therapeutic anticancer “vaccines” is the version stimulating the patient’s immune system to fight the disease [70,71]. The entry of microorganisms into the human body leads to the activation of immune mechanisms, which is manifested in increasing the number and recruitment of innate immune cells (especially neutrophils, monocytes/macrophages, and NK cells), the activation of cells of acquired immunity—that is, T and B lymphocytes—and the increased production of proinflammatory cytokines [72]. It is assumed that the immune system “mobilized” by bacteria is able to at least limit the development of cancer. This is especially important in cases where other treatments have failed. In addition, the therapeutic potential lies also in the products of bacterial secretion—toxins, enzymes, and bacteriocins. Their presence in the tumor environment can have a destructive effect on cancer cells. These and other versions are discussed in more detail below.

### 5.1. Scientists’ Favorite “Toys” in Looking for a Panacea

For more than one hundred years since Coley’s toxin, which was the first anticancer microbial “vaccine” based on a mixture of *Streptococcus pyogenes* and *Serratia marcescens*, many microorganisms have been studied for their antitumor activities (see Section 2). Some bacteria-based preparations were used, and continue to be used today, as a therapy complementary to standard treatment, increasing the patient’s chances of recovery.

Not every microorganism is suitable for the role of an anticancer “vaccine”. To be useful as an antitumor therapeutic agent, the bacterium should be: (1) Sufficiently invasive; that is, enter the tumor and survive and accumulate in it. (2) Safe for the patient—at least no more harmful than the cancer itself. To achieve this, it can use human commensal bacteria, which already live in the human body, or take pathogenic bacteria deprived of virulence factors (attenuated). (3) A microorganism that has completed its antitumor mission should be easily killed by an antibiotic—and, therefore, it should be sensitive to antibiotics, or should not easily acquire resistance to drugs. (4) The microorganism that can affect the tumor in different ways. It can be immunogenic, that is, it can stimulate a good immune response in a macroorganism, or it can produce some agents (toxins) that directly affect the tumor cells, killing them. Finally, the bacterium can be convenient for genetic manipulation to give it the desired properties—for example, the carrier of antitumor drugs.

For the long history of “bacterial anticancer therapy”, bacteria could be used both as a natural, wild-type strains and attenuated or genetically modified species; the last has been made for improving the natural ability of bacteria for the preferential colonization of tumors and creating a precisely controlled and highly specific nonpathogenic delivery shuttle.

Thus, for example, the bacillus Calmette–Guérin (BCG) is widely applied. BCG is a strain of *Mycobacterium bovis* which has been used for a hundred years as a tuberculosis vaccine. This microorganism—an etiological agent of bovine tuberculosis—was intentionally attenuated by many passages until it became an avirulent, but immunogenic, strain safe for human use. The links between the occurrence of tuberculosis and cancer regression were noted quite a long time ago, and in the last quarter of the 20th century, BCG was approved as the complementary treatment of bladder cancer. Treatment with the *M. bovis* BCG strain is considered effective when the intravesical infusion of the microbial suspension using urethral catheters is used [73,74].

The particular attention of researchers has long been attracted to anaerobic bacteria, such as *Clostridium* spp., *Salmonella* spp., and *Listeria* spp. The conditions inside the tumor are hypoxic, since the tumor is vascularized mostly from the outside. Therefore, the conditions inside the tumor are quite favorable for the development of obligate or facultative anaerobes. The greatest advantage of using these microorganisms is that they have a tendency to concentrate directly inside the tumor, in contrast to chemotherapeutics which spread throughout the body with the blood, also destroying normal, healthy cells [75].

The most common type of bacteria in use is probably *Clostridium*. The history of *Clostridium* as a fighter against cancer started in 1935, when an article was published describing the regression of advanced cancer under the use of sterile filtrates with *Clostridium histolyticum* [72]. *Clostridium* spp. is one of the largest prokaryotic genera, consisting of a heterogeneous group of Gram-positive, strictly anaerobic, spore-forming rods. Over the past decades, many studies have been conducted on the use of different clostridium species—*C. histolyticum*, *C. tetani*, *C. oncolyticum*, *C. beijenrickii*, and *C. novyi*—as tumor-targeting agents. One of the most promising is considered a genetically modified *Clostridium novyi-NT* strain. This strain can be attenuated by eliminating a residential phage-carrying α-toxin, which is responsible for toxicity. The spore-forming ability of clostridia is especially promising. Surviving as the spores in the oxygen environment, they germinate inside the tumor, causing hemorrhagic necrosis, cell lysis, and tumor regression. To date, the attenuated *C. novyi-NT* strain has positively undergone phase I and II of clinical trials [76,77]. A case was described where *C. novyi-NT* spores were intravenously injected into the patient with advanced leiomyosarcoma, which was followed by complete tumor regression [78,79]. Moreover, *C. novyi-NT* was reported to reduce glioblastoma growth induced in rats [80].

*Salmonella* sp. are Gram-negative, facultative anaerobic bacteria. They are able to move by flagella, facilitating their active migration from the vasculature into the deep tumor tissues. The most studied is *Salmonella enterica* serovar *Typhimurium* (*S. typhi*), an etiological agent of typhoid fever. In anticancer therapies, the attenuated strain of *S. typhi* VNP20009 is used for safety reasons, to avoid the dissemination into healthy organs such as the spleen or liver. VNP20009 is attenuated by the chromosomal deletion of the genes *purI* (purine) and *msbB* (lipid A). Clinical trials on the use of this microorganism for melanoma treatment began in 2002, and to date have gone through phase I. In addition, the VXM01 antitumor vaccine, which is based on the attenuated strain of *S. typhi*, has successfully passed phase I clinical trials [81,82,83].

Other bacteria, in addition to the above, were also recruited in anticancer therapy [84]. Among the Gram-positive bacteria, these are, in addition to clostridia, *Listeria monocytogenes*, lactic acid bacteria, bifidobacteria, and *B. subtilis*. Attempts are being made to employ some of these as direct anticancer agents, while others are to be used as vaccines or as vectors for drug delivery. Bifidobacteria and *Lactobacillus* are probiotic strains naturally found in the human gut. Oral administration of natural nonpathogenic *L. lactis* is used as a prominent therapeutic agent to treat inflammatory bowel disease and moderate ulcerative colitis. An extensive study on *Listeria monocytogenes* was focused mostly as a vaccine; moreover, some groups have used it as a gene-delivery vector for cancer therapy [85]. Among the Gram-negative bacteria following *S. typhimurium* is *E. coli*. *E. coli* strains are commonly nonpathogenic, in contrast to *S. typhi*, and can be found in the human gut. The latter makes it possible to use some *E. coli* strains without attenuation. It was shown that *E. coli* has the ability to colonize tumors in mice at a higher rate compared to normal tissues [86,87]. The most widespread probiotic strain in use is *E. coli* Nissle 1917, and also the wild-type MG1655 strain. Another Gram-negative species, *Pseudomonas aeruginosa* strain 1409, was exerted, as it was demonstrated to have a good therapeutic effect in the mouse TC-1 tumor model. It is worth noting, however, that this bacterium is highly pathogenic for humans and often highly resistant to basic antibiotics, so it is probably not very suitable for the direct treatment of cancer [88,89].

Not all mechanisms of the “anticancer” action have yet been adequately studied, but some are already known or suspected. They will be briefly discussed in the next section.

### 5.2. Mechanisms of Antitumor Action

Currently, several mechanisms of the destructive effect of microbes on tumors are being discussed. Regarding native or marginally modified bacteria, two main mechanisms are most often considered: stimulation of the immune response by bacteria and the production of cell-death-stimulating agents.

#### 5.2.1. Stimulation of the Immune Response

The immune system plays an important role in a tumor surveillance. Properly, abnormal cells are recognized and eliminated with the help of innate and adaptive immunity. The evolution of tumors occurs in some phases, namely in the elimination, equilibrium, and escape phases, when malicious cells become weakly immunogenic due to the development of tolerance and the immune system does not perceive them [90,91,92]. The immunotherapeutic strategy of cancer treatment supposes the stimulation of the immune system to destroy cancerous cells, and anticancer bacterial immunotherapeutic “vaccines”, respectively, propose to employ bacteria to enhance the antigenicity of tumor cells.

The key point in the induction of a systemic anticancer immune response is the ability of the immune system to recognize conserved pathogen-associated molecular patterns (PAMPs). Thus, the injection of any appropriate bacteria—killed or weakened—directly into the tumor has the potential to induce or restore the host immune response against the cancer. Moreover, the pioneer Coley, as well as some other researchers, tried applying not only intratumoral but also intravenous injections, resulting in durable cancer remissions in both cases—although, in general, it seems that intratumoral injections are considered more effective [25,93]. An even more rational way to increase the antigenicity of tumor cells is to try to create a vaccine based on isolated tumor-tropic microorganisms, as described in the previous sections.

The question is, which part of the complex and multilevel immune system does the bacteria interfere with to stimulate it? There are several works on which bacteria can stimulate the host’s immunity and target it in tumor cells, as well as the facts accumulated proving the involvement of a wide variety of immune-response mechanisms. As an example, consider one of the successful examples of the stimulation of the immune response by bacteria, the abovementioned BCG immunotherapy, which is considered as a gold-standard treatment for nonmuscle-invasive bladder cancer at a high risk of recurrence or progression. Preclinical and clinical studies have revealed that a robust inflammatory response to BCG consists of some steps, including: (1) the attachment of BCG; (2) the internalization of BCG into resident immune cells, normal cells, and tumor urothelial cells; (3) the BCG-mediated induction of innate immunity, which is orchestrated by a cellular and cytokine milieu; (4) the BCG-mediated initiation of tumor-specific immunity [74,94]. Getting into the bladder, BCG interacts with the epithelium; there, the activation of epithelial cells and antigen-presenting cells that produce cytokines and chemokines is observed. The appearance of macrophages, dendritic cells, lymphocytes, and neutrophils is also observed in the bladder wall. In vitro studies with the use of human NIMBC (nonmuscle-invasive bladder cancer) cancer cell lines showed that BCG increases the production of IL-6 and IL-8, GM-CSF (granulocyte–macrophage colony-stimulating factor), and tumor necrosis factor (TNF). Furthermore, data obtained from studies on human subjects showed that, after the introduction of BCG, neutrophils, macrophages, monocytes, T and B lymphocytes, and NK cells were present in the bladder, accompanied by increased levels of IL-1, IL-8, IL-15, IL-18, CXCL10 (CXC motif chemokine ligand 10), CC motif chemokine ligand (CCL)2, CCL3, and GM-CSF [74,95]. Neutrophils can directly affect tumor cells through their phagocytic activity and the ability to produce reactive oxygen species, the secretion of lytic enzymes, and factors inducing apoptosis, e.g., TRAIL (TNF-related apoptosis-inducing ligand). The role of NK cells in BCG-induced antitumor activity is not quite unclear, but the reduced efficacy of BCG immunotherapy in NK-cell-depleted mice compared with nondepleted mice, including both increased tumor growth and reduced survival, was reported. In the case of successful immunotherapy, the intensive secretion of IL-2, IL-12, IFN-, TNF-, and TNF- was observed, whereas, in the case of failure, the production of IL-4, IL-5, IL-6, and IL-10 was noticed [73,74].

In the work of Carroll et al. [93], another form of antitumoral immunotherapy, different from BCG, namely intratumoral injection of emulsified complete Freund’s adjuvant (CFA), was successfully used in mammary tumors in mice, in mastocytomas in mice and dogs, in equine melanomas, and also in human cancer patients. CFA, a known extremely potent immune stimulant, contains, in contrast to BCG, not alive, but heat-killed mycobacteria. All studies have shown major antitumor effects in a proportion of treated animals and safety for use in human patients. In general, it is obvious that the mechanism of immunotherapy by means of bacteria is extremely complex and still requires clarification.

#### 5.2.2. Production of the Cell-Death-Inducing Agents

There is a wide range of bacterial-derived products which can specifically target the cancer cells and exert a cytotoxic effect. Among the cytotoxic agents are bacterial toxins, enzymes, peptides, and some secondary bacterial metabolites.

<*Bacterial Toxins*>. Bacteria express and release some toxins that can be cytotoxic or, at lower concentrations, may result in the alteration of proliferation, differentiation, and apoptosis. Some of them are able to effectively inhibit tumor growth through cell-cycle arrest, tumor cell signal pathway interruption, and other mechanisms. One of the well-known antitumor agent bacterial toxins is cytolysin CylA. Cytolysins are pore-forming agents that generate multimeric pores in the cell membrane and contribute to cell destruction. Cytolysins are generally derived from *Escherichia coli*, *Staphylococcus aureus* [96], or *Salmonella typhimurium* [97,98]. Actually, mice treated with *S. typhimurium* or *E. coli* strains expressing the CylA toxin demonstrated tumor-growth inhibition [98,99]. Another well-known toxin is the diphtheria toxin (DT), which is a main virulence factor produced by *Corynebacterium diphtheria*. At low concentrations, this toxin has lethal activity on mammalian cells. Modified DT-based toxins have proved effective in the treatment of several types of cancers [100,101]. *Clostridium difficile* toxin includes two subtypes of cytotoxin and enterotoxin which can kill cancer cells by recruiting proinflammatory factors to activate the immune response [89]. *Clostridium botulinum* produces botulinum neurotoxin A, which reduces cell growth and proliferation in prostate cancer lines PC-3 and LNCaP [102], and induces apoptosis in breast cancer cell lines T47D [103,104]. Enterotoxin produced by *Clostridium perfringens* also has anticancer activity, which leads to dose-dependent acute toxicity by binding to the overexpressed claudin-4 receptor on pancreatic cancer cells [105]. Verotoxin 1 (VT-1), mostly recognized as Shiga toxin-1 (Stx1), is produced by pathogenic *E. coli* and can stop the cell cycle in the HCT116 cell line of colon cancer [106,107]. Exotoxin A (PE) synthesized by *Pseudomonas aeruginosa* inhibits protein synthesis through ADP ribosylation, leading to cancer cell apoptosis [107,108].

<*Bacterial Enzymes*> Bacteria produce a wide array of enzymes. Some of them can act on the essential amino acids required for tumor growth. One of the commonly investigated bacterial enzymes is L-asparaginase, an enzyme produced by *Escherichia coli*, *Bacillus subtilis*, Streptomyces, or Erwinia species [67]. This enzyme catalyzes the hydrolysis of asparagine, reducing its blood concentration and causing processes that are fatal to tumor cells [109]. L-asparaginase has been proven effective in the treatment of acute lymphoblastic leukemia, lymphosarcoma, neoplasia, and other malignancies [109,110,111]. Other bacterial enzymes of interest are arginine deiminase and arginine decarboxylase, which are involved in arginine catabolism [112]. It was demonstrated that arginine deaminase produced from *Streptococcus pyogenes* can consume arginine in tumor cells, resulting in decreased proliferation of arginine-deficient tumor glioblastoma multiforme [113].

<*Bacteriocins*> Bacteria produce a broad spectrum of ribosomal synthesized proteinaceous and peptide toxins—bacteriocins. These bacterial antimicrobial peptides are mainly directed against closely related bacterial species in the same niche. In some cases, bacteriocins can also inhibit the growth of tumor cells. The cationic charge and amphiphilic nature allow the bacteriocin to interact with the negatively charged cell membrane and destroy the membrane integrity, triggering the apoptosis of the cancer cells [114]. For instance, nisin, one of the well-studied and widely used bacteriocin produced by *Lactococcus lactis*, has demonstrated antimicrobial activity against the majority of the Gram-negative bacteria and has also demonstrated to be cytotoxic to MCF-7 cells (the human breast adenocarcinoma cell line) [115]. Another well-known class of antimicrobial peptides, colicins, also showed anticancer activity in in vitro studies [47].

<*Biosurfactant*> Biosurfactants are the surface-active substances of different structures produced by microorganisms. In recent years, it was reported that biosurfactants can act as anticancer agents by interfering with cancer-progression processes by inhibiting some crucial signaling pathways. In addition, biosurfactants can stimulate NK cells, inhibit angiogenesis, and induce apoptosis via death receptors in cancer cells [116,117]. Microbial biosurfactants are considered biodegradable and less toxic than synthetic analogs [118]. One of the examples is surfactin from *Bacillus safensis*, which exhibited pronounced antitumoral efficacy against T47D breast cancer cells and B16F10 mouse melanoma cells [119]. Another example is a cyclic lipopeptide, viscosin from *Pseudomonas libanensis*, which demonstrated extensive antitumor activities. The MTT results indicated that viscosin inhibited the proliferation of MDA-MB-231 in breast cancer. Moreover, viscosin inhibited the migration of the prostate cancer cell line PC-3M [120,121].

<*Dormant Spores*> In unfavorable conditions, bacteria survive by producing dormant spores. In the hypoxic tumor microenvironment, spores revive and transform into the active state. Thus, it was shown that *Clostridium novyi* NT spores are an effective therapeutic agent for experimental tumors in mice, and that they do not contain lethal toxins and do not cause any systemic side effects in the injected host [122,123].

Summing up the section, it is worth noting that, although bacteria produce a wide variety of therapeutics, the timely and effective release of these substances from bacteria into the microenvironment is an ongoing challenge. There have been attempts to solve this problem by expressing specific phage lysis genes [124] or lyse bacteria by means of antimicrobial drugs, and thus releasing plasmids for tumor cell uptake [125]. In addition to releasing therapeutics, instantaneous lysis can provide the release of bacterial adjuvants that may stimulate immune responses, also reducing population growth after completing its task.

Other mechanisms unrelated to immune stimulation or the production of tumor-destroying agents have also been considered. Particularly, bacteria are regarded as antitumor agents through the salvation of tumor cells by depleting required nutrients or through the formation of bacterial biofilms, which could play a role in carcinogenesis by inhibiting the growth, metastasis, and diffusion of tumor cells, and accumulating and delivering therapeutics [24,47,96]. However, whatever the mechanism of action of bacteria on the tumor affecting the macroorganism, it is worth keeping in mind that the main issue is the safety of such an approach. The principle of “do no harm” still remains fundamental in medicine; therefore, the possibility of complications in a patient subjected to “bacterial cancer therapy” should be minimized. Various approaches are used to ensure security, such as the weakening of bacteria, depriving them of pathogenic factors, as well as designing genetically engineered bacteria. The possibility to modify a bacterium or even almost create it anew, strengthening the desired properties and minimizing harm to humans, will be the subject of the final section.

## 6. Future Perspectives: Bacteriobots?

The term “bacteriobot”—bacterial microrobot—has been used in some articles to denote a new innovative theranostic methodology of bacteria-based fabrication for tumor therapy. However, in a more general sense, it can be used to refer to any bacterium that has been in any way intentionally modified [77,126,127]. It is worth noting that recent years are characterized by an explosive increase in the number of articles devoted to the creation of modified bacteria suitable for use as antitumor agents. This is due to the rapid development of genetic engineering methods; in particular, genome editing methods. Therefore, it is very likely to expect that, in the near future, we will observe a qualitative shift in this field of science. In this article, rather, retrospective, we did not set out to cover this issue in detail, so we will only touch on general trends. This topic is described in more detail in a number of excellent recent reviews [128,129,130].

One of the biggest challenges in cancer research is an efficient drug delivery to the tumor regions. To solve this problem, miniature devices would be suitable, characterized by active motion towards the tumor, penetration inside, and the accumulation in the tumor or nearby tissues. Such devices can be completely synthetic (chemically and/or physically actuated) containing only man-made materials, structures, and components, or cellular microrobots (biologically actuated) that exclusively consist of cell-made components and which are precision-engineered to exhibit anticancer effects. Moreover, hybrid versions consisting of both artificial and cell-made components that can be propelled by biological or artificial means (usually biologically actuated) can be constructed. The use of natural bacteria, particularly human commensals, as a vector for transferring a chemotherapeutic agent directly into the tumor could significantly reduce the side effects of treatment that usually accompany traditional chemotherapy [131,132]. Bacteria can reach the target passively by blood flow or more actively, for example, with the help of flagella. Bacterial flagella rotate in a propeller-like fashion and can achieve swimming speeds up to 300 μm/s. An attractive idea appears to be to use magnetic fields for guiding magnetic particles from their application site to the cancer, independently of the tumor localization. In this case, bacteria must be sensitive to magnetic fields. An interesting example includes magnetotactic bacteria, which contain naturally synthesized magnetic particles (magnetosomes), allowing them to sense magnetic fields and align their swimming directions along them [72,128]. Thus, *Magnetococcus marinus* (MC1) is a Gram-negative coccus found in the Atlantic Ocean. This microorganism has cilia arranged in two bundles located at one pole, which enable the bacteria to move (300 μm/s). Magnetosomes of this bacterium are special elements which are magnetite particles (Fe_3_O_4_) surrounded by membranes, forming chains in the cytosol. The presence of magnetite orients the bacteria with the Earth’s magnetic field. Using a powerful magnetic field, the same as in the MRI (magnetic resonance imaging) technique, it would be possible to direct bacteria containing magnetosomes to the tumor. Indeed, in a recent study, magnetic guidance led to significantly enhanced tumor accumulation of peritumorally injected MC1-based hybrid microrobots in living mice compared to nonguided MC1 bacteria [133,134]. Recently, magnetic-sensing *E. coli* was engineered in [135] by driving the formation of iron-rich bodies into bacterial cells.

Enhancing the tumor targeting is other important task that can be solved with the help of bacterial microrobots. As a result of mutations and other genetic/epigenetic aberrations, cancer cells and their microenvironments acquire chemical and physical characteristics that differ from normal cells. Specifically, they express cell surface proteins that can be divided into tumor-associated antigens, which are normal proteins being abnormally expressed by the cell, and neoantigens, which are novel, abnormal proteins. So, neoantigens represent unique targets, as they are only expressed by tumor cells. Genetic modification of the bacteria can help to display tumor-specific ligands on its surface. For example, the α_v_β_3_ integrin is overexpressed on cancer cells and a *Salmonella* ppGpp-deficient strain SHJ2037 was designed to display the integrin-binding peptide Arg-Gly-Asp (RGD) onto the bacterial surface to target the cancer cells. The SHJ2037 strain demonstrated a high tumor specificity and enhanced antitumor activity in MDA-MB-231 breast cancer cells and MDA-MB-435 melanoma xenografts with overexpressed α_v_β_3_ integrin [24]. Another approach is by genetically engineering bacteria to target tumor-associated antigens [24,136,137]. Thus, in the work of [137], it was demonstrated that *Salmonella* tumor specificity can be significantly improved via a surface-expressed single-domain antibody (VHH) directed to a tumor-associated antigen (CD20). Antibody-dependent bacterial targeting specified the infection of CD20+ lymphoma cells in vitro and in vivo while significantly diminishing nonspecific cell invasion.

**Figure 3:**
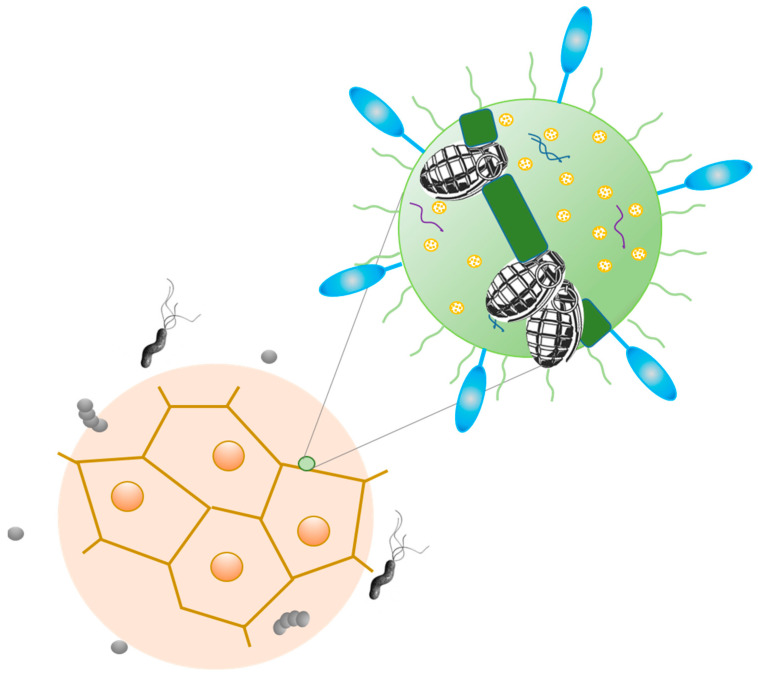
Bacteriobot—the bacterial microrobot, which is able to transcribe and translate the gene of interest, and encapsulate and precisely deliver to the cancer cells a wide range of chemotherapeutic and molecular drugs, antigens, toxins, and other therapeutics.

A multitude of reporter genes, cytotoxic proteins, and tumor-specific antigens can be expressed using genetically engineered bacteria as a vector. The engineered bacterium enters the target cells and expresses the transgene, resulting in the expression of therapeutic proteins (bactofection). The invasive bacteria can deliver genes intracellularly into the tumor cells, thus fostering bactofection into tumor cells, whereas the noninvasive strains are engineered to release therapeutic proteins external to the tumor microenvironment [24]. Many studies have confirmed the anticancer potential of engineered bacteria expressing death-inducers—ligands which penetrate into tumor cells and induce apoptosis [138,139,140]. Actually, the ideal bacterial microrobot should be a kind of “minicell”—the nanosized, anucleated, nondividing, and metabolically active cells that are able to transcribe and translate the gene of interest. Minicells should be able to encapsulate a wide range of chemotherapeutic and molecular drugs, si/shRNA, antigens, and therapeutic toxins to precisely deliver them to the cancer cells through the easy modification of the minicell surface with specific antibodies against receptor-targeted cancer cells [141] (Figure 3).

## 7. Conclusions

Bacteria are the constant companions of the human body throughout its life and even after its death. Before starting to write this review, having become interested in the idea of using human commensal bacteria to improve well-being after a serious disease, we did not imagine the scale of scientists’ desire to create an “ideal bacterium” possessing all the necessary properties—mobility, tumor tropicity, plasticity for modifications, as well as safety for humans.

Bacteria have many different tools for interacting with the tumor and with the host, and demonstrate a huge potential in cancer therapy. However, the clinical application of this kind of therapy still never became routine because of the possible uncontrollable adverse side effects. The greatest difficulty is to make the behavior of bacteria absolutely predictable. So far, bacteria are a double-edged sword in cancer therapy.

However, scientists have already advanced quite far on this pathway. Some genetically modified bacteria are already undergoing the last phases of clinical trials. The switch from natural, wild-type bacteria towards engineered analogues, their modifications as antitumor agents, antioncogenes, or immunogenic antigens, and their combination with other therapeutic processes improve their potential for cancer therapy. Some approaches involving the use of bacteria are used as auxiliary therapies for cancer. Further investigations and developments in the field of the bacterial therapy of tumors can provide new breakthroughs to cancer treatment.

## Figures and Tables

**Table 1:** Bacteria that are suspected to be associated with various types of cancer.

Microorganism	Cancer Type Associated	References
*Helicobacter pylori*	gastric carcinoma	[42,43]
*Salmonella enterica serovar Typhi*	gallbladder cancer	[48,49,50]
*Campylobacter jejuni*	small intestinal lymphomas, colorectal cancer	[51,52]
*Chlamydia psittaci*	ocular lymphomas	[52,53]
*Mycobacterium tuberculosis*	lung cancer	[54]
*Chlamydia pneumonia*	lung cancer, lymphomas	[53]
*Fusobacterium nucleatum*	colorectal cancer	[55,56]
*Bacteroides fragilis*	colorectal cancer	[57,58]
*Escherichia coli*	colorectal cancer	[57,58]
*Streptococcus bovis*	colorectal cancer	[59,60]
*Chlamydia trachomatis*	cervical cancer, lymphomas	[53,54,55,56,57,58,59,60,61]
*Porphyromonas gingivalis*	pancreatic and oral cancer	[62,63]

## Data Availability

Not applicable.
